# Hybrid Revascularization Approach Using Robot-Assisted Bilateral Internal Mammary Artery Grafting

**DOI:** 10.21470/1678-9741-2025-0008

**Published:** 2025-12-10

**Authors:** Hugo Monteiro Neder Issa, Luciano Matar, Andre Shuster, Guilherme Athayde, Leticia Ferreira, Martin Bucek, Pedro Romanelli, Diogo Ferrari Centenaro, Joana Ferreira Hornestam, Arthur Monteiro Neder Issa, David Glineur

**Affiliations:** 1 Division of Cardiac Surgery, University Ottawa Heart Institute, Ottawa, Ontario, Canada; 2 Division of Cardiovascular Surgery, Children’s Hospital for Eastern Ontario, Ottawa, Ontario, Canada; 3 Hospital Mater Dei, Belo Horizonte, Minas Gerais, Brazil; 4 Hospital Moinhos de Vento, Porto Alegre, Rio Grande do Sul, Brazil; 5 Research Institute, Children’s Hospital for Eastern Ontario, Ottawa, Ontario, Canada; 6 Pontifícia Universidade Católica de Minas Gerais, Belo Horizonte, Minas Gerais, Brazil; 7 Division of Cardiac Surgery, Memorial University, St. John's, Newfoundland, Canada

**Keywords:** Robotics, Coronary Artery Bypass, Bilateral Mammary Arteries, Coronary Artery Disease

## Abstract

Hybrid coronary revascularization combines minimally invasive surgical coronary artery bypass grafting with percutaneous coronary intervention. This case report describes a 72-year-old male with multivessel coronary artery disease treated using a hybrid approach: robot-assisted bilateral internal mammary artery grafting followed by percutaneous coronary intervention. This method leverages the strengths of both modalities, offering tailored treatment for specific coronary lesions. The patient’s postoperative course was uneventful, and follow-up demonstrated excellent outcomes.

## INTRODUCTION

**Table:** 

Abbreviations, Acronyms & Symbols	
BIMA	= Bilateral internal mammary arteries
CABG	= Coronary artery bypass grafting
CAD	= Coronary artery disease
CPB	= Cardiopulmonary bypass
HCR	= Hybrid coronary revascularization
ICS	= Intercostal space
LAD	= Left anterior descending artery
LIMA	= Left internal mammary artery
MAG	= Multiple arterial grafting
OM1	= Obtuse marginal 1
PCI	= Percutaneous coronary intervention
PL	= Posterolateral artery
RIMA	= Right internal mammary artery

Hybrid coronary revascularization (HCR) represents a tailored strategy for treating complex coronary artery disease (CAD), integrating surgical and percutaneous techniques to achieve optimal outcomes^[1]^. The approach uses arterial grafting, typically for the left anterior descending artery (LAD), combined with percutaneous coronary intervention (PCI) for non-LAD targets^[1]^. Robot-assisted coronary artery bypass grafting (CABG) further enhances this method, enabling minimally invasive harvesting of bilateral internal mammary arteries (BIMA) with precision and reduced recovery times^[2]^. This case highlights the feasibility and success of HCR, emphasizing the benefits of robotic assistance for surgical components and its technical aspects. Consent was obtained from the patient for this publication, and institutional Research Ethics Board approval was waived.

## CASE PRESENTATION

A 72-year-old male with a history of hypertension and dyslipidemia presented with stable angina. An echocardiogram revealed preserved biventricular function. Nuclear myocardial perfusion imaging demonstrated ischemia in the anterior and lateral coronary territories. Coronary angiography revealed severe multivessel disease, including significant stenosis of the distal left main proximal LAD, proximal obtuse marginal 1 (OM1), and posterolateral artery (PL). Fractional flow reserve assessment of the right coronary artery revealed a value > 0.8, excluding hemodynamically significant lesions.

### Preoperative Planning

After discussion with the heart team and the patient, a hybrid revascularization approach was selected. This involved robot-assisted CABG with BIMA grafting, with the right internal mammary artery (RIMA) anastomosed to the LAD and the left internal mammary artery (LIMA) grafted to the OM1, followed by PCI for the PL lesion.

### Patient Preparation

Patient preparation is critical for a successful robotic BIMA harvest. Under general anesthesia, a double-lumen endotracheal tube facilitates single-lung ventilation. The patient is positioned supine with a slight left chest elevation with a roll under the left scapula, ensuring optimal access to the left thoracic cavity. Both arms are secured alongside the torso. Defibrillator pads are placed on the right infraclavicular area and the left posterior chest. The groins are made available to access if needed, and the cardiopulmonary bypass (CPB) machine and a perfusionist are on standby during the procedure if required. The legs are exposed for potential saphenous vein harvest.

### Surgical Technique

#### Port Placement and Robotic Setup

In the left thorax, the ports are inserted. A 12 mm camera port is inserted in the fifth intercostal space (ICS) along the anterior axillary line, usually close to the nipple. With the camera inserted from the 12 mm port, two 8 mm instrument ports are placed, one in the third ICS and the other in the seventh ICS, forming a triangular layout. This configuration ensures the optimal maneuverability of robotic instruments. Special attention must be taken regarding the port inserted into the third ICS, as it may interact with the left shoulder and, therefore, can limit its range of movement. For this case, we also inserted in the fourth ICS a laparoscopic 5 mm port for vascular clip applier. The Da Vinci XI robotic system (Intuitive Surgical Inc., California, United States of America) is positioned on the patient's right side. With a 0-degree or 30-degree angulation, the camera provides high-definition, magnified views of the operative field. For most portions of the BIMA harvest, bipolar microtissue forceps are attached to the left robotic arm and spatula cautery to the right arm.

#### Right Internal Mammary Artery Harvesting

The RIMA is approached first, as a harvested LIMA would likely be damaged with the robotic instruments working on the RIMA bed. The left lung is deflated. Access to the RIMA bed involves creating a substernal plane extending to the right pleura. The right pleura is kept intact as much as possible to avoid right lung protrusion. Dissection begins by identifying the pulsating artery beneath the endothoracic fascia. The parietal pleura and fascia are carefully incised using monopolar cautery, exposing the RIMA along its length. The artery is skeletonized using sweeping movements of robotic instruments. The small branches are cauterized using a monopolar cautery spatula, or for large branches, we use the bipolar cautery micro forceps. The larger branches can also be clipped and divided with robotic scissors only with the Da Vinci Si, as the Xi has no clipping instruments. It is crucial to dissect and transect the proximal mammary vein to allow very proximal RIMA dissection. This can be performed with the help of a retractable spatula introduced under the xyphoid to push on the mediastinal fat close to the innominate vein. Video 1 shows the RIMA harvest.

**Video 1:**
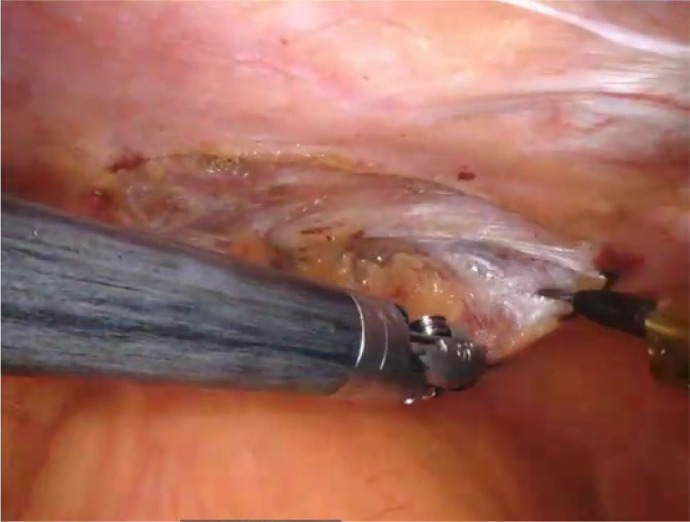
Right internal mammary artery harvested robotically. Link: https://youtu.be/q3Kdos5j2tw

#### Left Internal Mammary Artery Harvesting

The LIMA is dissected after the RIMA. The right lung may be fully ventilated, and the left may be ventilated at low volumes with CO2 inflation at 12 mmHg. The LIMA dissection mirrors the technique used for RIMA, employing the same robotic instruments and movements. Adjustments in port angulation or positioning may be required to optimize access. Heparin is administered once both mammary arteries are freed, and the distal end of them are clipped and divided. Once the mammary arteries are cut, they systematically have a torsion movement leading to a 360° twist. To avoid this, it is paramount to clip the distal end of the mammary on the mediastinal fat. Video 2 shows the LIMA harvest.

**Video 2:**
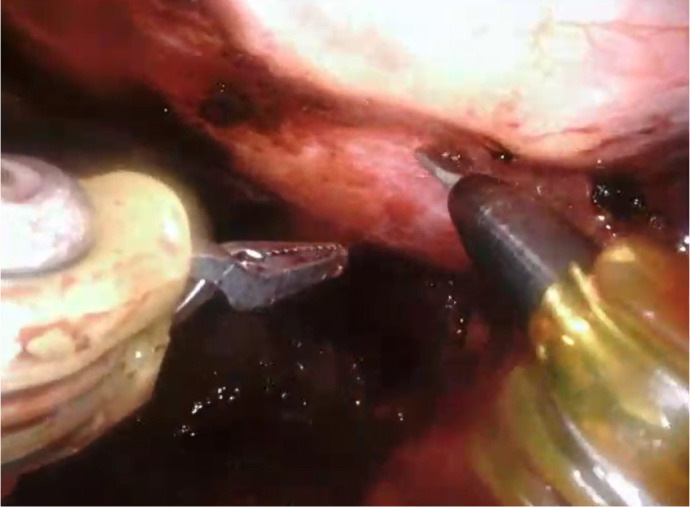
Left internal mammary artery harvested robotically. Link: https://youtu.be/ZIaAe90EA6g

#### Left Mini Anterolateral Thoracotomy for Coronary Graft Anastomosis

**Fig. 1:**
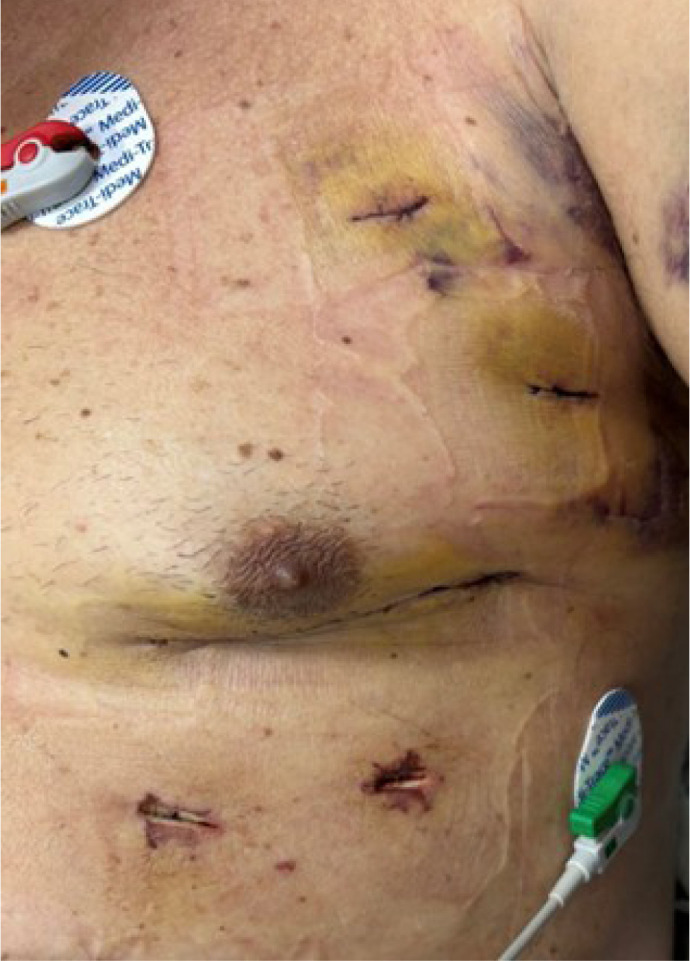
Postoperative incision and port insertion sites.

Once BIMA are harvested, a 4 - 6 cm long anterolateral thoracotomy is made (Figure 1), which usually englobes the 12 mm portal insertion incision. Before fully opening the ICS, a small hole is made in the ICS, and digital palpation is done to feel the apex of the heart, which indirectly shows us that we are in a good spot regarding surgical exposure. Usually, the fifth ICS is opened, but if necessary, the fourth or sixth ICS may be opened to achieve adequate surgical exposure. After opening the ICS, a mini-thoracotomy retractor is placed. The first step is the mammary recovery. Each mammary is exposed with two 6-0 Prolene® sutures. One on each side of the mammary to avoid any twist. The flow in the mammary is accessed. The pericardium is then opened longitudinally.

The next step is the distal anastomoses. Blood pressure should be brought up to allow manipulation of the heart with hemodynamic stability. We aim for a systolic blood pressure of 140 - 150 mmHg. The sequence of distal anastomoses is dictated by the surgeon’s preference and the potential degree of ischemia in each territory. We routinely start with LAD anastomosis.

The Octopus® NUVO (Medtronic, Minnesota, United States of America) adequately exposes and stabilizes the coronary target. Complementarily, pericardium stay sutures may be placed to optimize coronary exposure in special for the lateral and inferior walls. The Octopus® NUVO is applied using the 6 mm incision at the sixth/seventh ICS. The stabilizer holder is fixed on the table arms to obtain maximal stabilization. CPB can be used with femoral cannulation in cases of inadequate exposure of the target vessels or hemodynamic instability. If the patient presents hemodynamic stability with adequate coronary exposure, we proceed to the distal anastomosis off-pump. For coronary bleeding control, a temporary suture is placed around the coronary artery to be grafted, proximally to the planned arteriotomy. This occludes the coronary for a short period and allows better visualization. A blower is also used to improve the visibility of the coronary. After arteriotomy, an intracoronary shunt can be placed, and the suture around the coronary artery may be removed. Then, the distal anastomosis is performed with a 7-0 or 8-0 Prolene® suture with standard instruments. We check all bypass grafts with a Doppler flow probe. Protamine is administered after confirmation of adequate graft flow and hemostasis.

By the end of the procedure, a drain is inserted in the left pleural space via the incision where the 8 mm portal for the left robotic arm was placed. The left lung is reinflated, and the proper lie of the grafts and the chest tube should be checked during lung reexpansion. The ICS is reapproximated, and the subcutaneous tissue and skin are closed. Intercostal nerve infiltration with anesthetic drugs is an option to optimize immediate postoperative pain control.

#### Procedural Considerations

Carbon dioxide insufflation pressures are maintained between 6 and 12 mmHg throughout the procedure to enhance visibility. Blood pressure and saturation are closely monitored to avoid hemodynamic instability, especially during port placement, RIMA harvest, and pleural insufflation. Conversion to sternotomy is an option in cases of inadequate visualization, bleeding, or hemodynamic compromise. The safety and effectiveness of the procedure must never be jeopardized by the minimally invasive nature of the robotic approach. Adequate training and experience with robotic systems are essential for surgeons performing these procedures to minimize risks and complications.

### Postoperative Course

The patient’s recovery was uneventful. He was extubated within six hours postoperatively. We routinely start aspirin 81 mg within two hours after surgery and clopidogrel within six hours. On the second postoperative day, the patient underwent PCI in the PL balloon angioplasty, followed by a successful stent implantation. The surgical distal anastomosis was checked, and angiography confirmed widely patent grafts (RIMA to LAD and LIMA to OM1). The patient was discharged on postoperative day four and remained symptom-free at short-term follow-up.

## DISCUSSION

This clinical case underscores several essential considerations in managing complex CAD using a robotic-assisted HCR approach.

The use of multiple arterial grafting (MAG), as demonstrated in this case with BIMA, offers superior outcomes compared to single arterial grafting. MAG is associated with improved long-term graft patency, reduced rates of myocardial infarction, and better overall survival^[3]^.

The HCR employed in this case provides distinct advantages, including the ability to tailor revascularization strategies to patient-specific anatomy and disease complexity. Combining robotic-assisted CABG with the PCI approach reduces the invasiveness of treatment while ensuring comprehensive revascularization^[4]^. However, hybrid procedures require meticulous coordination between surgical and interventional teams, adding complexity to perioperative planning and execution.

Robotic-assisted CABG, particularly for BIMA harvesting, offers several benefits over conventional sternotomy approaches. The robotic technique minimizes surgical trauma, reduces infection risk, accelerates recovery, and enhances quality of life, making it particularly advantageous for high-risk patients. High-definition visualization and precise robotic instruments enable meticulous dissection and skeletonization of both mammary arteries, preserving their integrity for optimal graft function^[5]^. Despite these advantages, the robotic approach demands significant training and experience to mitigate technical challenges and ensure patient safety.

Notably, this case marks a milestone in cardiac surgery in Brazil as the index robotic-assisted cardiac procedure performed in Belo Horizonte and likely the index robotic-assisted BIMA harvest associated with a hybrid approach in the country. This achievement reflects the advancing capabilities of cardiac care in the region and sets a precedent for adopting minimally invasive techniques and hybrid procedures in complex coronary revascularization.

## CONCLUSION

This case illustrates the feasibility, safety, and clinical benefits of an HCR approach augmented by robotic assistance. The combination of robotic precision, the advantages of MAG, and a tailored hybrid strategy highlights a paradigm shift toward less invasive yet highly effective management of multivessel CAD.

## Data Availability

The authors declare that the data supporting the findings of this study are available within the article.

## Artificial Intelligence Usage

The authors declare that no artificial intelligence tool was used in the preparation of this article.
